# Factors Affecting Medication Adherence in Children Receiving Outpatient Pharmacotherapy and Parental Adherence

**DOI:** 10.1111/jcap.12113

**Published:** 2015-05-19

**Authors:** Masaharu Nagae, Hideyuki Nakane, Sumihisa Honda, Hiroki Ozawa, Hiroko Hanada

**Affiliations:** ^1^ Department of Nursing Nagasaki University Graduate School of Biomedical Sciences Nagasaki Japan; ^2^ Department of Psychiatric Rehabilitation Sciences Nagasaki University Graduate School of Biomedical Sciences Nagasaki Japan; ^3^ Department of Community‐Based Rehabilitation Sciences Nagasaki University Graduate School of Biomedical Sciences Nagasaki Japan; ^4^ Department of Neuropsychiatry Nagasaki University Graduate School of Biomedical Sciences Nagasaki Japan

**Keywords:** Children and adolescent, medication adherence, outpatient

## Abstract

**Problem:**

Although pharmacotherapy is an effective treatment for many psychiatric disorders in children and adolescents, medication adherence rates among children are low. This study clarifies factors affecting children's medication adherence and the role of parental involvement.

**Method:**

Patients aged 7–17 years with a history of psychotropic medication treatment and their mothers were included in this study. Each mother and child completed self‐administered questionnaires. Thirty mother–child pairs who fully completed the questionnaires were included in the analysis.

**Results:**

Medication adherence was greater in children and mothers when mothers felt that “children's symptoms improved with treatment and medication.” Medication adherence in children and mothers significantly correlated with the child's reported trust in their parent.

**Conclusions:**

The results suggest the need for psychosocial support that considers the influence of mothers on medication adherence in children treated in child psychiatry departments.

In general, child and adolescent psychiatric patients are treated with a combination of psychosocial interventions and pharmacotherapy. For many diseases or severities of illness, guidelines recommend pharmacotherapy as the first line of treatment because of its high therapeutic efficacy. The use of psychotropic medications among children with psychiatric problems can improve not only psychological symptoms, but also quality of life, academic performance, and relationships with friends, family, and others (Hamrin, McCarthy, & Tyson, 2010). Despite these benefits, children generally exhibit lower rates of medication adherence. Without adequate treatment, these patients are at risk for more severe psychiatric symptoms, declining academic performance, interpersonal problems, suicide, and family stress (Hamrin et al., 2010). Children's medication adherence rates vary between 13.2% and 89.8%, and the majority of studies demonstrating high adherence rates adopt a child or caregiver self‐assessment (Adler & Nierenberg, 2010; Hamrin et al., 2010). However, adherence rates assessed through self‐report are likely to be higher than actual adherence rates (Pappadopulos et al., 2009; Yang et al., 2012).

In recent years, many studies have acknowledged the willingness of children to take medications. For instance, Floersch et al. (2009) conducted interviews with adolescents about their subjective experiences with psychotropic medication therapy and found that although youth believed in the need for and effectiveness of psychotropic medication, they had many questions about how these medications work and substantial concerns about side effects. Floersch et al. (2009) suggested that these results occurred because of a large gap in the experience and knowledge of youth regarding the effects and side effects of medication. Additionally, Kranke, Floersch, Kranke, and Munson (2011) conducted interviews with adolescents about their subjective experiences with psychotropic medication therapy, and found an association between taking medications and self‐stigma. Specifically, many adolescents had experienced stigma and shame related to their condition and therefore preferred not to disclose their use of psychotropic medication. In another study, Brinkman et al. (2012) interviewed adolescents aged 13–18 years with attention‐deficit hyperactivity disorder (ADHD). Here, it was demonstrated that when trying to understand their symptoms and the necessity for medication, adolescents frequently compared their mental condition with and without medication. Townsend, Floersch, and Findling (2009) demonstrated the effectiveness of evaluating adolescent patients' adherence to psychotropic medications with the Drug Attitude Inventory‐30 (DAI‐30), which evaluates patients' subjective attitudes about medication. However, evaluating patient adherence to antidepressants or stimulants using the DAI‐30 is insufficient because this measure was specifically designed to assess the attitudes of adults with schizophrenia who take antipsychotic medications. Altogether, children and adolescents seem to recognize the importance of psychotropic medications, and medication adherence in child and adolescent psychiatry should be assessed in terms of willingness. The various factors affecting this willingness to take psychotropic medications should be more closely examined.

The factors influencing children's medication adherence include the following: (a) presence of comorbidity (e.g., ADHD with depression, ADHD with anxiety disorder, and schizophrenia with depression increase adherence; ADHD with oppositional disorder or borderline personality disorder decrease adherence); (b) particular prescriptions and dosages; (c) adverse reactions to medications; (d) social stigma about psychiatric treatment or psychotropic medication; and (e) children's understanding of the reasons for taking medication (Charach & Gajaria, 2008; Hamrin et al., 2010; Lloyd et al., 1998). Further interacting with these factors to affect adherence are family issues, each child's individual issues, and the nature of specific treatments. For instance, in one study, Charach, Volpe, Boydell, and Gearing (2008) identified predictors of adherence to psychostimulants in children with ADHD and found four significant factors: the children, their parents/families, the healthcare professionals, and the prescribed medications.

Previous studies have revealed that parental awareness of treatment risks and benefits is closely related to children's adherence (Hoza, Johnston, Pillow, & Ascough, 2006). Furthermore, both children's knowledge about the reasons for taking medication and their parents' views on medications affect the continued use of medications (Thorell & Dahlström, 2009). Since parental consent is necessary for children to receive medical treatment, parents select their children's treatments. It is natural, therefore, that parental awareness of their children's symptoms and treatments can significantly affect the course of treatment (Brinkman et al., 2012). While children are young, their parents are actively involved in their medication management. However, as the children grow older, parents gradually transfer responsibility for medication management to them. During adolescence, many children begin to manage their own medication and are more in control of continuing psychopharmacology. Young adolescents, however, may have different views about taking the medications than their parents. For example, adolescents differ from their parents in their beliefs about ADHD and their attitude about medication use (Charch, Yeung, Volpe, Goodale, & dosReis, 2014), and adolescents are less willing to treat their ADHD with medications than their parents (Bussing et al., 2012).

In order for children to continue receiving appropriate treatments, we must improve adherence by addressing the factors affecting child and adolescent willingness to take psychotropic medications. In particular, it is important to assess the effects of parental adherence and attitudes and how these relate to children's adherence. Furthermore, it is necessary to clarify any age‐related differences in the influence of parents. In this study, we examined the factors affecting medication adherence among both mothers and their children in child and adolescent psychiatry departments as well as the associations between maternal and child medication adherence.

## Method

### Participants

Outpatients and their mothers visiting a child and adolescent psychiatry or pediatrics department in Nagasaki Prefecture were included in this study. Five hospitals collaborated on this research. All patients were taking psychotropic medications upon diagnosis according to the International Statistical Classification of Diseases and Related Health Problems, 10th Revision (ICD‐10). Since there is no advanced practice nursing system in Japan, all patients had been prescribed the psychotropic medication by physicians. Exclusion criteria were being younger than 7 or older than 18 years of age, illiteracy, severe autism, and having an IQ (intelligence quotient) of less than 70. Thirty‐seven mother–child pairs were referred to study researchers by their attending doctors, and 33 of these pairs consented to participate in the study.

### Procedure

A survey of mothers and their children was conducted concurrently using a mailed questionnaire. It was necessary to mail the questionnaire to respondents because there were instances where only the child or the mother visited the hospital. This survey was conducted over a period of 2 months from August to September 2009. The aims and ethical considerations of this study were explained prior to participation, and written consent to participate in the survey was obtained from both the mother and the child. Ethical approval for the study was obtained from the Ethics Committee of the University where the researchers were employed.

### Materials

#### Demographic and Clinical Characteristics

Along with several questionnaires, the survey consisted of questions about participants' demographics and clinical characteristics. Children were asked six items, including age, gender, name of attending physician, name of medication administered, reasons for medication, and if they had received an explanation about the medication. Mothers were asked seven items, including age, family composition, history of psychiatric treatment, treatment history with psychotropic medications, child's history of clinic visits, the relationship between changes in their children and the effects of medications following clinic visits, and if they were close to one or more individuals who were taking psychotropic medications. The researchers also interviewed patients' attending physicians for further information on children's psychiatric diagnoses and medications.

#### Child Adherence Questionnaire

The Child Adherence Questionnaire (CAQ) is a scale created by Nagae, Honda, and Hanada (2011) based on the Drug Attitude Inventory‐10 (DAI‐10) (Hogan, Awad, & Eastwood, 1983) to measure adherence in children receiving pharmacotherapy with psychotropic medications. The scale consists of two factors (“attitude toward medication” and “awareness of medication influences”) with a total of 25 items to which individuals can respond either “YES” or “NO.” Positive and negative answers are given a score of +1 and −1, respectively. Total scores on the scale are then calculated, with higher scores indicating better adherence. Total scores range from −25 to +25 points. Scores for “attitude toward medication” and “awareness of medication effects” can range from −10 to +10 points and from −15 to +15 points, respectively. Previous research has demonstrated Cronbach's alpha coefficients of .76 for the CAQ's total score, .59 for the “attitude toward medication” subscale, and .69 for the “awareness of medication influences” subscale (Nagae et al., 2011).

#### Mother's Adherence Questionnaire

Nagae, Honda, and Hanada (2010) created the Mother's Adherence Questionnaire (MAQ) to measure adherence in parents whose children are treated with psychiatric pharmacotherapy. The MAQ consists of 22 items: 10 derived from the DAI‐10 (Hogan et al., 1983) about their children's medication, and 12 related to factors affecting the mother's adherence. Respondents are required to answer questionnaire items with either “YES” or “NO.” Positive and negative answers are given a score of +1 and −1, respectively. The total score is then calculated, with a higher score indicating better adherence. Scores range from −22 to +22, and the instrument's Cronbach's alpha coefficient is .72 (Nagae et al., 2010).

#### Questionnaire for Parent–Child Mutual Trust

This Japanese scale was created by Sakai, Sugawara, Maeshiro, Sugawara, and Kitamura (2002) to measure and bidirectionally evaluate parent–child interpersonal trust based on respondents' expectations of and trust in a parent or child. This measure consists of one questionnaire assessing a parent's trust in their child and another assessing a child's trust in his/her parent. There are paternal and maternal versions of each questionnaire. As this study sought to investigate mother–child relations, the maternal versions were adopted. Each questionnaire is composed of eight items rated on a scale of 1 to 4. For children, the scale options were 1 = *Disagree*, 2 = *Slightly disagree*, 3 = *Slightly agree*, and 4 = *Agree*. For mothers, the response options were 1 = *Very unlikely*, 2 = *Slightly unlikely*, 3 = *Slightly likely*, and 4 = *Very likely*. A higher score indicated greater trust. The range of possible scores is from 8 to 32. In previous research, Cronbach's alpha coefficients of .89 for the children's maternal trust questionnaire and .85 for the maternal trust in their children questionnaire were obtained (Sakai et al., 2002).

### Data Analysis

Of the 33 mother–child pairs who consented to participate, 30 completed all questionnaires with no omissions and were included in data analyses. After a simple tabulation was conducted on participants' demographics, the medians and quartiles of participants' CAQ and MAQ scores were calculated in relation to their demographic characteristics. Then Mann–Whitney *U* test and Kruskal–Wallis test were performed. Additionally, Spearman's rank correlation coefficients were calculated for the MAQ, CAQ, and trust scales. Statistical analyses were conducted using PASW statistics 18.0. All tests were two sided and employed a significance threshold of 5%.

## Results

### Sample Characteristics

Participants' ages (19 boys, 11 girls) ranged from 7 to 17 years (*M* = 12.9 years, *SD* = 2.7). The most prevalent ICD‐10 F9 diagnosis code group was “Behavioral and emotional disorders with onset usually occurring in childhood and adolescence” (nine cases). Antidepressants were the most commonly prescribed medication (17 cases). Three patients reported that they did not know about their prescriptions (i.e., the name and reason for taking the prescribed medication), and five responded that they had not received any explanation about their medications from healthcare professionals. Patients' mothers were approximately 40 years of age. Seven mothers had a personal history of visiting psychiatric clinics and nine had a treatment history with psychotropic medications. Moreover, eight mothers answered that they had family member(s) other than the children enrolled in the study taking psychotropic medications. Of all the mothers survey, 25 acknowledged that their children's symptoms had improved after psychiatric clinic visits and 23 recognized that their children's improvement was due to the effects of the medication(s) they had been prescribed.

### Relationships Between CAQ or MAQ Scores and Participant Characteristics

The medians and quartiles of participants' CAQ and MAQ scores were calculated according to demographic and clinical characteristics, and Mann–Whitney *U* test and Kruskal–Wallis test were conducted (Table 1). Children of mothers who answered “YES” to the question “Did you notice an improvement in any of your child's symptoms after visiting the clinic?” scored higher on the CAQ than did children of mothers who answered “NO” (*p* = .065). Further, children of mothers who answered “YES” in response to the question “Do you think your child's improvement is due to his/her taking the medication(s)?” scored higher on the CAQ than did children whose mothers answered “NO” (*p* = .086). Mothers scored higher on the MAQ when they answered “YES” to the following items: “Did you notice an improvement in any of your child's symptoms after visiting the clinic?” (*p* = .042), “mother's treatment history with psychotropic medications” (*p* = .061), “Do you have any family member(s) taking psychotropic medications other than the relevant child?” (*p* = .056), and “Did you think there was an improvement in any of your child's symptoms after he/she took the medications?” (*p* = .061). Common factors contributing to greater CAQ and MAQ scores were “mothers' recognition of improvements in their children's symptoms after visiting the psychiatry department” and “mothers' acknowledgement of their children's improvement as the result of the effects of prescribed medication(s).”

**Table 1:** Associations Between Participant Characteristics and the CAQ/MAQ (*n* = 30)

Item	Category	*n*	CAQ scores		*p*	MAQ scores		*p*
			Median (Q1–Q3)			Median (Q1–Q3)		
Children's background								
Gender^a^	Male	19	11	(7, 15)	.158	11	(7, 17)	.210
	Female	11	9	(−3, 13)		7	(3, 15)	
Age (years)^b^	Primary school	10	9	(3.5, 12)	.712	11	(8, 19)	.503
	Junior high school	14	12	(0, 15)		9	(4, 15.5)	
	High school	6	9	(6, 10.5)		9	(4.5, 14)	
Family composition^a^	Living with grandparents	8	6	(−2.5, 13)	.256	13	(6.5, 19)	.219
	Nuclear family	22	9	(8.5, 15)		9	(5, 15.5)	
	Living with parents	18	9	(0, 15)	.692	9	(5, 15.5)	.285
	Single‐mother household	12	10	(5.5, 13)		12	(5.5, 18.5)	
	Sibling(s)	20	9	(−0.5, 14.5)	.397	9	(5, 17)	.397
	Only child	10	10	(8.5, 13.5)		11	(8.5, 15.5)	
Mother's background								
Age (years)^b^	Younger than 39	11	9	(7, 15)	.972	11	(9, 19)	.549
	40–44	10	10	(0, 13.5)		10	(−0.5, 17.5)	
	45–49	7	9	(−3, 15)		9	(5, 15)	
	50 or older	2	9	(9, 9)		8	(5, 11)	
Mother's history of visiting psychiatric clinics^a^	Yes	7	7	(5, 15)	.666	13	(11, 19)	.061*
	No	23	9	(3, 13)		9	(5, 15)	
Mother's treatment history with psychotropic medications^a^	Yes	9	11	(8, 15)	.283	11	(9, 14)	.476
	No	21	9	(0, 13)		9	(4, 18)	
Do you have any family member(s) taking psychotropic medications other than the relevant child?^a^	Yes	8	8	(5.5, 14)	.801	12	(11, 18.5)	.056*
	No	22	9	(2.5, 13.5)		9	(4.5, 15.5)	
Did you notice an improvement in any of your child's symptoms after visiting the clinic?^a^	Yes	25	9	(8, 14)	.065*	11	(6, 17)	.042**
	No	5	−3	(−6, 11)		5	(−5, 10)	
Do you think your child's improvement is due to his/her taking of medication(s)?^a^	Yes	23	9	(9, 15)	.086*	11	(7, 17)	.061*
	No	7	3	(−3, 13)		5	(−5, 13)	
Basic information about child's treatment								
Medical history (months)^b^	Less than 1 year	3	15	(−3, 21)	.366	13	(5, 19)	.747
	1–3 years	10	8	(−1.5, 13)		12	(1, 19)	
	3 or more years	17	9	(8, 14)		9	(5, 13)	
Prescription (multiple answers available)								
Antipsychotic medication^a^	Yes	9	9	(3, 14)	.476	9	(4, 16)	.594
	No	21	9	(6, 14)		11	(8, 17)	
Antidepressant^a^	Yes	17	9	(−2, 14)	.320	8	(5, 15)	.281
	No	13	9	(8, 14)		11	(9, 17)	
Central nervous system stimulant^a^	Yes	7	11	(9, 15)	.226	11	(9, 19)	.386
	No	23	9	(1, 13)		9	(5, 15)	
Recognized the attending physician's name^a^	Yes	23	9	(3, 13)	.701	11	(5, 17)	.774
	No	7	9	(9, 15)		9	(1, 17)	
Understanding of medication^b^								
Knowing the name(s) of and understanding the reasons for taking medication		16	10	(7, 13)	.125	9	(5, 14)	.518
Knowing the name(s) of but not understanding the reasons for taking medication		6	9	(9, 19.5)		14	(7.5, 17.5)	
Not knowing the name(s) of but understanding the reasons for taking medication		5	15	(0, 18)		9	(0, 16)	
Not knowing the name(s) of or the reasons for taking medication		3	−1	(−9, 5)		19	(−5, 19)	
Received an explanation about the medication from medical staff^a^	Yes	25	9	(7, 14)	.229	11	(5, 16)	.957
	No	5	−1	(−12, 14)		9	(0, 19)	

*p < .1, **p < .05.

a The Mann–Whitney *U* test was used for two groups.

b The Kruskal–Wallis test was used for three or more groups.

CAQ, Child Adherence Questionnaire; MAQ, Mother's Adherence Questionnaire; Q1, 1st quartile; Q3, 3rd quartile.

### Correlations Among Scales

Spearman's rank correlation coefficients among the MAQ, CAQ, and mother–child trust scale are shown in Table 2. For participants under 14 years of age, a statistically significant correlation was found only between the attitude factor of the CAQ and MAQ scores (*r* = .562, *p* = .037). For participants 14 years of age and over, a statistically significant correlation was found between CAQ and MAQ scores (*r* = .724, *p* = .002). Moreover, both the “attitude toward medication” and the “awareness of influences of medication” factors of the CAQ correlated with MAQ (respectively *r* = .535, *p* = .033; *r* = .612, *p* = .012). A statistically significant correlation was found between children's mother–child trust scale scores and both MAQ scores (*r* = .544, *p* = .029) and CAQ scores (*r* = .619, *p* = .011). No statistically significant correlations were found between mothers' mother–child trust scale scores and either MAQ or CAQ scores.

**Table 2:** Difference Due to Age in Correlations Among Scales

	CAQ	CAQ subscale		Scale assessing trust between mother and child	
		Attitude toward medication	Awareness of influences of medication	Child's trust in his/her mother	Mother's trust in her child
Age < 14 (*n* = 14)					
MAQ	0.294	0.562*	0.080	0.347	0.058
CAQ				0.283	−0.109
Attitude toward medication				0.351	−0.058
Awareness of influences of medication				0.200	−0.127
Age ≧ 14 (*n* = 16)					
MAQ	0.724**	0.535*	0.612*	0.544*	0.347
CAQ				0.619*	0.430
Attitude toward medication				0.600*	0.360
Awareness of influences of medication				0.417	0.331

*p < .05, **p < .01; Spearman's rank correlation coefficient.

CAQ, Child Adherence Questionnaire; MAQ, Mother's Adherence Questionnaire.

## Discussion

### Factors Affecting Children's Medication Adherence

We found no statistically significant difference between CAQ scores and participant characteristics (Table 1). However, analyses conducted with a 10% significance threshold suggested that potential factors influencing children's adherence were “mothers' recognition of improvements in their children's symptoms after visiting the psychiatry department” and “mothers' acknowledgement of their children's improvement as the result of the effects of prescribed medication(s).” The finding that children's adherence was associated with maternal awareness of the pharmacotherapy is consistent with the results of other studies (Hoza et al., 2006; Thorell & Dahlström, 2009). However, unlike previous studies, we found no association between adherence and participant characteristics such as gender, age, and living with parents. It is likely that these factors were not found in the present study because of our relatively small sample size.

Five children reported that they “did not receive any explanation about the medication they were prescribed from healthcare professionals,” and three shared that “they did not know much about their prescriptions” (i.e., the name and reasons for taking the medication). We must be careful when interpreting this. It is likely that these children had received some explanation about the medication when prescribed; however, they may not have understood the doctor or may have forgotten the explanation. In general, patients' understanding and acceptance of the need for treatment and medication is strongly associated with adherence (Sabate, 2003). In our survey, however, we noted no significant associations between these previously mentioned factors and medication adherence.

One reason for the lack of significant associations may be the result of the substantial participant age range. In a literature review of pediatric psychotropic medication adherence, Hamrin et al. (2010) reported that parents exert greater influence on the adherence of younger children than on adolescents. Furthermore, this review indicated that younger children exhibit better psychotropic medication adherence than older children. According to Piaget's (1970) theory of cognitive development, children 7–11 years of age become capable of logical thought under concrete circumstances, yet cannot engage in hypothetical or abstract thought. Only after age 11 can children think about abstract concepts, use deductive reasoning, and engage in logical and methodical problem solving. Fitting this model, Floersch et al. (2009) found that among 12‐ to 17‐year‐olds, adolescents taking psychotropic medications wanted to know the reasons for taking medication, the medication's effects and adverse effects, and how to manage their medication. Charch et al. (2014) suggested that incorporating input from young adolescents when making clinical decisions could potentially improve the continuity of ADHD treatment. Thus, in response to changes in children's awareness of medical regimens and mental and social growth, healthcare professionals need to provide adolescent patients with the necessary knowledge about their prescribed medications. Overall, they must enable children to acquire more knowledge about their treatment.

### Factors Affecting Mothers' Adherence to Their Children's Medication

To date, no study in child and adolescent psychiatry has evaluated mothers' adherence to the treatment of their children or the factors affecting this adherence. However, parental awareness of a child's pharmacotherapy can have a profound effect on medication adherence and attitudes toward treatment (Brinkman et al., 2012; Charach et al., 2008; Hoza et al., 2006; Thorell & Dahlström, 2009). The only factor affecting maternal adherence that was clearly found in this study was “mothers' recognition of improvements in their children's symptoms after visiting the psychiatry department” (Table 1). Other possible influential factors were “mothers' history of visiting psychiatric clinics,” “the presence of close person(s) taking psychotropic medications,” and “mothers' acknowledgement of their children's improvement as the result of the effects of prescribed medication(s).”

Our evaluation of parental adherence and potentially influential factors suggests several implications for both research and practice. Parents visit hospitals with the hope that their children's health will improve. Stigma about psychiatric treatment or pharmacotherapy, however, could cause parents to become critical of their children's treatment (Larson & Corrigan, 2008). Medical adherence is known to be influenced by how patients understand the effects of their treatment and form negative beliefs about the treatment (Sabate, 2003). Similarly, in this study, “actual feelings regarding the effects of psychiatric treatment,” such as “mothers' acknowledgement of their children's improvement as the result of the effects of prescribed medication(s),” were suggested to positively contribute to mothers' adherence. Additionally, adherence was higher in mothers who answered “YES” to questions concerning whether they had “a history of visiting psychiatric clinics” or “close person(s) taking psychotropic medications.” Possible explanations for this result are that mothers with these experiences have less resistance toward treatment with psychotropic medications or more knowledge about pharmacotherapy. Thus, they may have fewer negative beliefs regarding treatment or feel less stigma about their children being treated with psychotropic medications.

One effective intervention that could enable mothers to positively influence their children's treatment is to provide mothers with sound psychoeducation about their children's psychological symptoms and pharmacotherapy (Ferrin & Taylor, 2011; Lucksted, McFarlane, Downing, & Dixon, 2012). If mothers gain enough knowledge about their children's disorder or treatment, they may better understand the importance of their child's treatment. In addition to reducing stigma, interventions that integrate peer support can produce other benefits such as promoting the development of the skills needed to work with their children, problem‐solving abilities, and access to social supports (Lucksted et al., 2012; Shihabuddeen & Gopinath, 2005). In these ways, mothers' adherence to children's medication can be further improved.

### Relationships Between Medication Adherence in Mothers and Children

In general, parents are responsible for supervising their children's treatment and medication use. Thus, many previous studies have implemented parental educational interventions. In this study, among adolescents aged 14 and older, we observed a statistically significant correlation between CAQ and MAQ scores. Both CAQ components (“attitude toward medication” and “awareness of influences of medication”) correlated with MAQ. However, under 14 years of age, we found a statistically significant correlation only for the attitude factors of CAQ and MAQ. This suggests that children under 14 and their mothers may disagree in their awareness of the influences of medication. This could be the result of children under 14 being unable to convey the influences of medication to their mothers. Older children may be more able to convey and communicate their attitudes and awareness of the effects of medications. We estimate that mothers may begin transferring control of medication management to children when the children are around 14 years old. Moreover, because young children's attitudes toward medication are affected by their mothers' adherence, interventions improving mothers' understanding of the need for medication can attenuate stigma and improve child adherence, even when the child is older and more in control.

The present study did not investigate the extent to which mothers involved their children in the management of their medication. This should be clarified in future research. When children were 14 and older, their adherence and mothers' adherence were both correlated with their trust in their mothers, but not with mothers' trust in their children. Consequently, this suggests that children's medication adherence may be improved if maternal adherence is improved, e.g., by using a targeted educational intervention. Furthermore, this effect may be further enhanced if children's trust in their mothers is increased. For instance, Fristad, Goldberg‐Arnold, and Gavazzi (2003) improved family interactions and perceptions of parental support as well as parental knowledge about childhood mood symptoms by providing group psychoeducation to children with mood disorders and their families. It would also be necessary to educate children in order to establish stronger mother–child relationships. Specifically, mothers could build their children's trust by working alongside them on their treatments.

### Nursing Implications

In nursing, the focus is on the whole patient as opposed to the doctor's focus on the disorder and medical procedures. Nurses assess how the life of the patient has been affected by disorder and treatment and provide care accordingly. In particular, in the United States, advanced practice nurses (APNs) are major prescribers of psychotropic medications, and they tend to adapt regimens to patient preference and family and environmental situations, rather than simply giving a prescription or instruction (Gilliss & Mundinger, 1998). The results of this study suggest that for children taking psychiatric medications a careful negotiation with both the parent and the improvement of the parent–child relationship are imperative for APNs to influence the family and initiate or sustain medication treatment. In Japan, APNs are not allowed to prescribe medications, but a registered nurse could collaborate with other medical professionals to achieve this goal.

Previous studies have recommended a multimodal intervention rather than relying on one single intervention (Drake et al., 2015). This study indicated treatment outcomes may be improved by developing a comprehensive intervention program that includes providing both the parent and the child with knowledge, improving adherence of both parent and child, improving the relationship between parent and child, and promoting peer support. In this spirit, we have conducted a group psychoeducation program focused on promoting adherence among children and parents in cooperation with other medical professional that will be the subject of a future article.

### Limitations

A major limitation of this study was its relatively small sample size of 30 mother–child pairs. This research lacked detailed information regarding past treatments and adherence (e.g., whether participants had received some psychoeducation about their medication, whether children had actually received an explanation about their medication when it was prescribed). However, our results suggest several fundamental ways that medication adherence could be improved in child and adolescent psychiatry. The accumulation of a more extensive body of research is needed to enable further in‐depth investigation.

## Conclusion

The results of this study suggest two possible factors affecting children's medication adherence. Specifically, these included “mothers' recognition of improvements in their children's symptoms after visiting the psychiatry department” and “mothers' acknowledgement of their children's improvement as the result of the effects of prescribed medication(s).” Additionally, other possible influential factors were “mother's history of visiting psychiatric clinics,” “the presence of close person(s) taking psychotropic medications,” and “mothers' acknowledgement of their children's improvement as the result of the effects of prescribed medication(s).” A statistically significant correlation was found between children and mothers' adherence; in particular, children's “attitude toward medication” was strongly correlated with mothers' adherence. While children's adherence correlated with levels of trust in their mothers, it did not correlate with their mothers' trust in the children. The results of this study may be useful in designing nursing interventions to support children and adolescents receiving psychiatric treatment with psychotropic medication. Future studies that include a more detailed examination of the factors affecting medication adherence using a larger sample are required, as well as those that assess differences between children and adolescents.
